# Sociomoral Reasoning Skills during Childhood: A Comprehensive and Predictive Approach

**DOI:** 10.3390/brainsci12091226

**Published:** 2022-09-10

**Authors:** Frédérick Morasse, Annie Bernier, Gabrielle Lalonde, Sébastien Hétu, Miriam H. Beauchamp

**Affiliations:** 1 Department of Psychology, Université de Montréal, Montreal, QC H3C 3J7, Canada; 2 Centre de Recherche du CHU Sainte-Justine, Montreal, QC H3T 1C5, Canada

**Keywords:** sociomoral reasoning, children, long-term predictors, social cognition

## Abstract

Sociomoral reasoning (SMR) is an essential component of social functioning allowing children to establish judgments based on moral criteria. The progressive emergence and complexification of SMR during childhood is thought to be underpinned by a range of characteristics and abilities present in the preschool years. Past studies have mostly examined concurrent associations between individual factors and SMR. Using a more comprehensive and predictive approach to identify early predictors of school-age SMR would contribute to a more complete picture of SMR development. This study aimed to investigate the contribution of four domains of preschool predictors to SMR at school-age: demographic (age, sex, parental education), cognitive (executive and sociocognitive functions), behavioral (internalizing and externalizing behaviors), and familial (parent–child interactions, parental stress) factors. Parents of 122 children 3 to 5 years (M = 3.70, SD = 0.66 years, 51% girls) completed questionnaires and children were administered executive and sociocognitive tasks. Parent–child interactions were assessed using an observational approach. SMR was measured four years later using the SoMoral task. A four-step hierarchical regression analysis revealed that executive functions and internalizing problems were significant independent predictors of SMR. These findings provide a more comprehensive understanding of the early precursors of SMR during childhood.

## 1. Introduction

Childhood is characterized by the development of a range of social skills essential to adequate social competence [1,2,3]. These include sociomoral skills such as sociomoral emotions, behaviors, decision-making, and reasoning. Sociomoral reasoning (SMR) refers to the ability to analyze social situations and establish judgments based on moral criteria including notions of right and wrong [4,5]. SMR develops gradually throughout childhood, evolving from an egocentric perspective to a socio-centric one that allows children to consider others’ intentions and beliefs and to incorporate moral concepts in their reasoning such as justice, respect, and responsibility [6,7,8]. This progression is thought to be sustained by a range of child characteristics and functions (e.g., age, sex, cognitive skills) or environmental factors (e.g., socioeconomic status [SES], family functioning) [9,10,11], and by the maturation of the social brain network [12,13,14]. Individual and environmental characteristics in the preschool years are known to shape children’s future social experiences and overall development and are associated with social competence in the long-term [15,16,17,18]. Yet, most studies to date have investigated the contribution of these factors to SMR in silos, rather than comprehensively, and have mainly considered concurrent associations. Thus, identifying the early childhood predictors of school-age SMR is particularly relevant given that sound social competence, including adequate SMR, is crucial for creating and maintaining relationships and adopting prosocial behaviors [1,19,20]. 

Most of the pediatric literature documenting SMR contributors has focused on characteristics or functions within four broad domains: (i) demographic characteristics; (ii) cognitive functions; (iii) behavioral; and (iv) familial factors. 

**Demographic characteristics** include SES and age, previously found to be associated, both cross-sectionally and longitudinally, with SMR [6,21,22]. Differences in SMR based on biological sex have also been highlighted in several studies [6,22,23]. Together, the results of these studies suggest that more mature SMR is generally observed in older children, in girls, and in children from more affluent families or with higher levels of parental education [9,22,24]. 

**Cognitive functions** underlie how individuals process information and think about the world [25]. Among these, executive functions (EF) and sociocognitive abilities appear to play an important role in SMR by providing information about intentions and motivation, allowing children to consider others’ perspectives in their reasoning and inhibit their self-interest in order to make a decision that is beneficial for the greater good [8,9,26,27]. More specifically, better EF such as inhibition, planning, flexibility, and reasoning have been associated with more mature SMR skills [9,22,23,28]. In their cross-cultural study, Cowell and colleagues [22] reported that the concomitant association between EF and SMR in children aged 5 to 12 years holds across five regions (Canada, China, Turkey, South Africa, USA), supporting the robustness of the association across cultures. Further, evidence from a longitudinal study indicates that EF are concurrently associated with SMR at 6 years, and significantly predict SMR at 9 and 12 years [23]. 

Sociocognitive functions have also been linked to SMR as illustrated by concomitant associations with sympathy [23,24], empathy [29,30], affect understanding or recognition [11,31], theory of mind (ToM) [22,32], and SMR. In addition, using a longitudinal design, Daniel and colleagues [23] found that sympathy for others’ distress assessed at 6 years of age predicted SMR three years later. A similar study conducted by Lane and colleagues [33] showed that ToM skills at 2.5–4 years predicted SMR two years later. Thus, both EF and sociocognitive functions seem to contribute to SMR skills throughout childhood. However, few studies have examined their associations longitudinally, especially across preschool and school-age when the early building blocks of social skills become especially important to the establishment of more complex social interactions and quality peer relations.

**Behavioral factors** underlie a child’s reaction to a given situation and can be divided into internalizing (e.g., emotional regulation, anxiety) and externalizing behaviors (e.g., aggression, anger) [34]. Aggressive and antisocial behaviors have been associated with poorer SMR skills [35,36]. A study with preschoolers reported that those with higher levels of anger or anxiety provided fewer socio-centric justifications for moral transgressions [37]. Hinnant and colleagues [9] found that 10-year-old children with greater emotional regulation abilities presented better SMR skills. Despite these reported associations between SMR and both externalizing and internalizing behavior, their predictive power needs to be further investigated in order to better understand their link with SMR at school-age. 

**Familial factors** are widely recognized as determinant in children’s social functioning, especially in the preschool years, when the family environment constitutes the main context for social interactions [38]. As such, higher levels of SMR have been found in children whose mothers display warmer interactions [39], use more guidance and explanation in their reasoning [40] and show greater parental support [41]. In addition, numerous studies report associations between mother–child relationship quality, represented by cooperation, maternal responsiveness, and shared positivity, and child SMR [9,10,42,43,44]. For instance, Kochanska [10] found that mother–child relationship quality, assessed between the ages of 9 and 22 months, predicted children’s SMR skills at 56 months of age. Children who experienced positive interactions with their mother expressed more internalized and mature SMR in social situations. Other studies focusing more broadly on children’s social functioning suggest that higher levels of parental stress are associated with poorer social skills in children (e.g., they are less cooperative, prosocial, and socially mature) [45,46,47]. Together, these studies suggest that the family in which children grow up, as well as the quality of parental relationships and interactions, shapes the development of their SMR skills.

While previous research has emphasized the importance of SMR building blocks during childhood, most focus on single or limited factor domains [23,40,41] or on a specific developmental period, using cross-sectional designs [9,22]. For instance, Augustine et al.’s study [40] reveals the predictive contribution of behavioral and environmental factors assessed at age 2 on the SMR of 5-year-olds, but does not consider cognitive or familial predictors. Hinnant and colleagues [9] provide a more comprehensive model, including EF, emotional regulation abilities, maternal education, and mother–child cooperation, but only study these relations concurrently at 10 years. Given that SMR evolves significantly between preschool (3–5 years) and school-age (6–11 years) [6,48] and appears to be associated with a broad range of factors, this study aimed to investigate the contribution of demographic characteristics, cognitive functions, behavioral, and familial factors assessed during preschool to SMR at school-age. It was expected that each domain of factors would provide a significant incremental contribution to the prediction of child SMR. 

## 2. Materials and Methods

This study is part of a larger prospective longitudinal research project that aimed to investigate cognitive and social outcomes after early childhood traumatic brain injury. The original study followed a group of children who sustained traumatic brain injury and two typically developing comparison groups (community controls without injury and children with minor orthopedic injuries) over a five-year period during which data were collected at four time points. At each time point, children completed a 3-h neuropsychological assessment including cognitive, sociocognitive, and behavioral tasks, and parents completed questionnaires regarding their child’s functioning. For the purpose of this sub-study, the current sample included only children from the two typically developing comparison groups. The groups were combined given that previous work showed no differences on over 40 demographic or cognitive variables (e.g., parental education, ethnicity, intellectual and executive abilities, behavior) [49,50]. Candidate predictors were selected from the variables available when the children were 3 to 5 years of age and SMR outcome was assessed approximatively four years later. This study was approved by the local research ethics committee. Parents provided informed written consent prior to participation and were compensated $30 at each time point for their child’s participation.

### 2.1. Participants

The current sample includes 122 typically developing children (*M*_age_ at predictors assessment = 3.70, *SD*_age_ = 0.66 years; 62 girls; 97% Caucasian children) and their primary caregiver (111 mothers). Typically developing children without injury were recruited via information pamphlets distributed in local daycare centers, whereas typically developing children with minor orthopedic injuries (i.e., limb trauma leading to a final diagnosis of strain, laceration, simple fracture, contusion or unspecified trauma to an extremity) were recruited in an urban tertiary pediatric emergency department between 2011 and 2015. For all children, exclusion criteria were: (i) clinical diagnosis of any significant congenital, neurological, developmental, metabolic, or psychiatric condition; (ii) less than 36 weeks of gestation; (iii) history of traumatic brain injury; and (iv) child and parent not fluent in French or English. Children who were diagnosed with any of the exclusion conditions (e.g., autism spectrum disorder, traumatic brain injury) during the study were excluded from the sample. A total of 21 participants were excluded between initial contact and predictor assessment (the most frequent reason was that they obtained a clinical diagnosis or sustain a brain injury during that period).

### 2.2. Measures

#### 2.2.1. Demographic Characteristics 

The primary caregiver completed an in-house questionnaire documenting child age and biological sex as well as parental education, which was used as a proxy for SES and was obtained by averaging both caregivers’ educational qualifications on an 8-level scale ranging from doctoral degree (8 points) to less than seven years of school (1 point). When educational information was available for only one person (e.g., single caregiver household) an individual score was used. 

#### 2.2.2. Cognitive Functions 

**Planning.** Welsh’s simplified version of the Tower of Hanoi task [51,52] consists of a planning task in which children are asked to recreate an arrangement of discs in a minimum number of moves, by moving one disc at a time and always placing smaller discs over larger ones. Children have to reproduce six configurations and one point is allocated for each correct arrangement (maximum of 6 points). This task has excellent test-retest reliability (*r* = 0.90) [53]. A higher score indicates better planning abilities. 

**Flexibility.** The Conflict Scale [54] is a categorization task consisting of four levels of increasing difficulty that assesses cognitive flexibility. Children are asked to categorize items (e.g., plastic animals, cards) according to a certain rule, which changes after six trials. For example, children are first asked to play “the color game”, where they have to sort the items by color. After six trials, they are informed by the experimenter that they are now going to play the “shape game”, where they have to sort the items by shape instead of color. Each level consists of 12 trials, for a maximum of 48 points. The test-retest reliability for this task is excellent [55]. A higher score suggests better cognitive flexibility. 

**Inhibition.** The Shape Stroop [43,56] is an adaptation of the classic Stroop task assessing inhibition. First, children are presented with six fruits (three large and three small) and asked to identify each fruit and its size (e.g., small apple, big banana) to verify that they have the basic knowledge required for the task. Then, they are presented with three different cards depicting a small fruit embedded in a large fruit (e.g., a small banana in a large apple) and are asked to point to each small fruit (e.g., “show me the small banana”). Children must therefore inhibit an automatic response (i.e., the large fruit) to provide a more controlled one (i.e., the small fruit). One point is allocated for each small fruit correctly identified, for a maximum of three points. A higher score indicates better inhibition. Given that performances on the Shape Stroop appears to follow the same trajectory as inhibitory control development measured by a validated questionnaire, the task has previously been deemed appropriate to measure inhibition [57]. 

**Theory of mind.** The Desires task [58] assesses children’s understanding of how a character’s desires (fulfilled or not) can affect their feelings. Children are presented with two stories describing a character’s search for a desired object in a particular location. For each story, three different endings are presented: (1) The character finds the desired object; (2) the character finds nothing; (3) the character finds a different object. After each ending, they are asked “does the character feel happy or sad?”. One point is awarded for each correct answer and the sum (ranging from 0 to 3 points) was used in analyses, where a higher score indicates better theory of mind. The Desires task is moderately associated with other ToM measures and similar associations with external variables have been found, suggesting adequate construct validity [58,59].

#### 2.2.3. Behavioral Factors

**Child behavior**. The preschool version of the Child Behavior Checklist [CBCL 1.5–5 years, 34] is a parent report questionnaire documenting behavioral problems on two main scales: (a) Internalizing problems, including Emotionally Reactive, Anxious/Depressed, Somatic complaints and Withdrawn subscales; (b) Externalizing problems, including Attention problems and Aggressive behavior subscales. For each item, the primary caregiver rates their child’s behavior using a scale ranging from 0 (not true) to 2 (very true or often true). Internal consistency for both internalizing (α = 0.88) and externalizing problems (α = 0.87) subscales is good. The total raw scores for the two main scales were used as predictors. A higher score for both internalizing (0–72 points) and externalizing (0–48 points) scales indicates more behavioral problems.

#### 2.2.4. Familial Factors

**Parenting stress.** The Parental Stress Index—Brief PSI, [60] is a self-report questionnaire assessing the distress experienced by the parent with regard to their relationship with their child as well as their parental role. The 12-item parental distress subscale is used to document more specifically how the caregiver feels in their role as a parent (e.g., competent, restricted, depressed, supported). Each item is rated on a 5-point Likert scale from 1 (strongly agree) to 5 (strongly disagree) for a total score ranging from 0 to 60 points. Internal consistency for the current sample was good (α = 0.88). The total raw score was used, where a higher score indicates more difficulty adjusting to parenthood and higher levels of stress related to the parental role. 

**Quality of parent–child interactions.** The Mutually Responsive Orientation scale MRO, [61,62] is an observational measure that focuses on the dyadic nature of parent–child exchanges and can be used to assess the quality of parent–child interactions. In this study, parents were asked to interact with their child for 10-min periods in two different contexts (snack and toy-centered activity). Each period was videotaped and later scored on three subscales (Harmonious Communication, Mutual Cooperation, Emotional Ambiance) by trained assistants. For both periods of parent–child interactions, an MRO score ranging from 1 to 5 was computed by averaging the three subscales (two-rater intraclass correlation coefficient: MRO-Snack = 0.80 and MRO-play = 0.87). The two resulting MRO scores were then averaged to create a total MRO score (ranging from 0 to 5 points), where higher scores indicate more cooperative, harmonious, and emotionally positive interactions between parent and child, and lower scores indicate more disconnected, unresponsive, hostile, and affectively negative interactions. 

#### 2.2.5. Main Outcome

**Sociomoral reasoning**. The children’s version of the visual, computer-based Socio-Moral Reasoning Aptitude Level task (So-Moral) was administrated [6,63]. This task has age and gender-specific versions and includes nine sociomoral dilemmas presenting a conflict centered on sociomoral domains (e.g., concerns with justice, welfare/harm and rights) using first-perspective pictures of real child actors. Each scenario is presented using an introductory screen, three pictures depicting a sociomoral conflict and a question representing a dichotomous decision (e.g., would you cheat or would you not cheat) see Figure 1 in [6]. Following their decision, children are asked to provide a verbal justification that is later scored by two trained assistants using a standardized coding system [64] adapted from a cognitive-developmental approach to SMR [8,65]. SMR scoring was based on the following stages: (1) Centration and authoritarian-based consequences; (2) egocentric/pragmatic exchanges; (3) interpersonal focus; (4) societal regulations; and (5) societal evaluation. Transition stages (e.g., 1.5, 2.5) were used to account for answers that provide elements of two consecutive SMR stages. Justifications presenting elements of non-consecutives stages were coded according to the highest schema detected. The SMR score (0 to 45 points) was obtained by summing the nine justification scores (intraclass correlation coefficient = 0.89). 

### 2.3. Statistical Analyses

Statistical analyses were performed using SPSS 27.0 software. Due to challenges associated with attrition in this longitudinal study, there were missing data on some predictor variables (1–21%) and the main outcome four years later (43%). Little’s MCAR test was used to analyze the pattern of missing data and suggested that they were missing completely at random (*p =* 0.16). Given that Little’s test can lack power [66], further analyses were conducted to investigate whether complete and incomplete cases differed on any of the study variables. When compared to those with available data, participants with missing data on the Conflict Scale (n = 5) had lower scores on the Tower of Hanoi (*t* = −2.4, *p* = 0.02); participants who did not complete the Tower of Hanoi (n = 9) had lower scores on the MRO (*t* = −3.6, *p* = 0.001); participants with missing data on SMR (n = 53) had lower scores on the Desires task (*t* = −2.4, *p* = 0.02). Missing data were imputed on all study variables with incomplete cases using the multiple imputation procedure available in SPSS. Twenty-five imputed data sets were generated using all study variables as well as sociodemographic data. Following best-practice recommendations (Enders, 2010), analyses were performed on each imputed data set, and results across the 25 imputed data sets subsequently pooled. 

Prior to main analyses, the relations among the executive function variables (i.e., planning, flexibility and inhibition) were investigated. Intercorrelations between the three variables were found (*r* = 0.33, *p* < 0.001; *r* = 0.30, *p* < 0.001; *r* = 0.14, *p* = 0.08). They were submitted to a principal component analysis that revealed a one-factor solution (Eigen values > 1.0) representing 51% of the total variance. A principal axis rotation (oblimin) was then applied and the three variables loaded on the same factor—flexibility (0.80), planning (0.68), inhibition (0.65)—suggesting that a general executive factor was the best fit for the data [67]. A composite score for executive functioning was therefore computed by averaging the three scores. The composite score ranges from 0 to19 points and was used in further analyses. 

A hierarchical regression analysis was conducted to examine the predictive power of the four domains of factors assessed between 3 and 5 years on SMR measured four years later. Demographic characteristics were entered in the first block, followed by cognitive functions in the second block, behavioral factors in the third block, and familial factors in the fourth block. Results corresponding to *p* < 0.05 were considered statistically significant. The strength of correlations and effect sizes were determined according to Cohen’s criteria [68]. 

## 3. Results

### 3.1. Descriptive Results and Correlations 

Participants’ demographic characteristics and task performance are presented in Table 1 and predictor variable correlations with the main SMR outcome are presented in Table 2. Participants’ mean total score for SMR was 18.63 out of a possible 45 points, which corresponds to the second stage (e.g., egocentric/pragmatic exchanges) on the standardized coding system. 

### 3.2. Hierarchical Regression 

Results of the hierarchical regression analyses are presented in Table 3. In the first block, demographic characteristics (sex, age, parental education) did not contribute significantly to the model (F (3,112) = 1.10, *p* = 0.41). Cognitive functions (EF and ToM) were added in the second block and explained a significant 13% of the variance in SMR (∆F (2,110) = 8.69, *p* = 0.001) with a medium effect size (f2 = 0.16). Introducing behavioral factors (internalizing and externalizing problems) in the third block did not explain a significant portion of additional variance in SMR (∆F (2,108) = 2.84, *p* = 0.06). Finally, familial factors (parental stress and quality of parent–child interactions) were introduced in the fourth block and did not explain additional variance in SMR (∆F (2,106) = 0.31, *p* = 0.74). The overall model including all variables was significant (F (8,107) = 2.99, *p* = 0.003) and explained 21% of the total variance (f2 = 0.27) in SMR, with EF and internalizing problems as significant independent predictors, indicating that children with higher executive functioning and more internalizing problems showed higher levels of SMR.

## 4. Discussion

This study aimed to investigate the contribution of demographic characteristics (age, sex, parental education), cognitive abilities (EF and ToM), behavioral (internalizing and externalizing problems), as well as familial factors (parental stress and quality of parent–child interactions) measured in preschool to SMR four years later. Study hypotheses were partially confirmed. The cognitive domain was the only one that independently predicted SMR. When all predictors were taken into account simultaneously, better EF and more internalizing behaviors were the only independent predictors of more mature SMR. 

With respect to SMR, children on average provided sociomoral justifications based on a concept of pragmatic deals or exchange of favors with others, corresponding to the second stage of SMR according to the cognitive-developmental approach [6,63,65]. The SMR stage observed in this study is consistent with that expected for early school-age children, whose SMR is more likely to be based on authority and consequences and becomes more flexible and context-centered during this period. However, although school-age children are able to recognize that people can have a different understanding of a situation and progressively consider elements of interpersonal relationships, their SMR is often mainly focused on self-interest [6,11]. For example, in a situation where children have the opportunity to cheat to win a game, they might choose not to do so despite wanting to win, simply because they do not want their friends to stop playing with them. Thus, the decision is ultimately based on personal benefits (i.e., I don’t want to stop playing or lose my friends) and not on how others might feel (i.e., my friends will be sad). 

The significant contribution of EF found in this study is in line with previous work investigating the predictive role of EF in child SMR [9,22,69]. However, whereas past studies have mostly focused on this predictive relation during the school-age period, when EF are more complex and sophisticated [70,71], the present findings suggest that even emerging EF provide a foundation for later SMR. This could indicate that children who have good EF during early childhood more easily self-regulate their behavior, inhibit their own point of view in favor of that of others’, and consider multiple elements in their reasoning (e.g., people’s perspectives and intentions, social context), enabling them to interpret social situations in a more nuanced way from a young age, which could lead to greater SMR maturity later in childhood. 

Internalizing problems were identified as an independent predictor of SMR. Children whose parents reported higher levels of internalizing problems between 3 and 5 years old had better SMR skills four years later. Although this association between more problems and greater skills is somewhat counterintuitive, it is consistent with past studies suggesting that behavioral traits such as being more withdrawn, shy, anxious, or fearful, are not necessarily associated with poorer social skills [39,72,73,74,75]. Indeed, children with non-clinical levels of shyness and fearfulness might be more observant in social situations, more aware of social cues and intentions [76,77,78,79], as well as more intrinsically motivated to do the right thing to avoid social transgressions [61,75,80]. With respect to SMR, children exhibiting these internalizing behavioral traits during early childhood might understand the meaning of social cues and the social consequences associated with a course of action (e.g., peer rejection following social transgression) more easily. In later childhood, this could manifest itself by a level of SMR based more on social benefits than fear of authority or other consequences. 

Finally, counter to study hypotheses, some domains did not significantly contribute to SMR. Although demographic characteristics have been previously associated with SMR, they did not appear to contribute in this particular sample. Other studies using similar methods have also failed to document such a relation [28,30]. Given the methodological limitations associated with imputed data in this study, child age was represented by their age at the time of predictor assessment rather than at SMR assessment, which could in part explain why age did not significantly contribute to SMR in the current model. In addition, few families came from disadvantaged backgrounds, possibly offsetting putative detrimental effects of lower SES on social competence [81]. With respect to familial factors, most studies have reported an indirect association with SMR, by which parental influence is moderated by child individual characteristics (e.g., emotional regulation skills, cognitive functioning, temperament) [9,10,39,40]. Interaction effects were not tested in this study due to lack of statistical power, therefore there was no opportunity for these relations to emerge from the data. Furthermore, it is also possible that the selected measures did not capture the proximal familial factors most likely to be related to school-age SMR, such as parental SMR skills or sociomoral values [82,83,84,85]. 

This study is one of the first to use a comprehensive range of factors paired with a longitudinal design to better understand the early predictors of SMR in school-age children. Nonetheless, certain limitations have to be considered to properly interpret the findings. First, although the important amount of missing data in the initial data set were handled using best-practice recommendations, some key variables, such as age at SMR assessment could not be imputed. Second, given the modest sample size and limited statistical power, the number of predictors had to be restricted and interaction effects were not tested. Third, the homogeneous composition of the sample, both in terms of socioeconomic status and ethnicity, limits the generalizability of the results. Fourth, although inclusion criteria extended to both official languages of the country, it is possible that a selection bias was induced with respect to parental education/SES, given that allophone families, who are not fluent in either French or English, might be more financially disadvantaged. Parent report questionnaires were completed only by the primary caregiver, introducing possible parental bias. Although this study was conducted with a longitudinal design, the present findings do not inform on causal relationships and can only be understood in terms of statistical associations. Future studies using larger and more diverse samples as well as additional variables (e.g., empathy, temperament, parental SMR) and their interactions would be beneficial. Given that relationships between children and their two parents tend to differ, involving both caregivers in questionnaire completion and parent–child interactions would provide a more extensive understanding of child and family functioning.

## 5. Conclusions

This study aimed to determine what demographic, cognitive, behavioral and familial factors contribute to SMR in the long-term. Findings indicate that children with better EF or more internalizing behaviors during the preschool period display more mature SMR at school-age. Although other domains did not independently contribute to SMR, adding them to a comprehensive model sheds light on possible latent associations that are not accounted for in studies focusing on a single predictor or domain. Future studies with larger sample could use multivariate analyses to explore the inter-relations between the predictors of each domain. Furthermore, the lack of significant results for some of the four domains should not be considered a deterrent to using a comprehensive approach in future studies, but rather an opportunity to build on the present efforts to identify the complex relations underpinning SMR during childhood. Overall, the current study provides a basis for future efforts to establish even more comprehensive models of sociomoral development in both healthy and clinical samples.

## Figures and Tables

**Table 1:** Demographic characteristics of study sample.

Variable	Frequency	%
**Sex**		
Male	60	49.2
Female	62	50.8
**Ethnic origin**		
White/Caucasian	97	79.5
Black/African-American	4	3.3
Hispanic	3	2.5
Asian	2	1.6
Other	9	7.4
Missing data	7	5.7
**Maternal education**		
Doctoral degree	7	5.7
Master’s degree	37	30.3
Bachelor’s degree	60	49.2
College	12	9.8
High school graduate	4	3.3
Incomplete high school	1	0.8
Missing data	1	0.8
**Paternal education**		
Doctoral degree	6	4.9
Master’s degree	36	29.5
Bachelor’s degree	41	33.6
College	18	14.8
High school graduate	11	9
Incomplete high school	2	1.6
Missing data	8	6.6

**Table 2:** Sample characteristics and main variable outcomes.

Variable	M	SD	Correlation with SMR (*r*)
**Demographic characteristics**			
Age at predictor assessment	45.74	7.91	−0.04
Parental education	6.12	0.86	0.16 ^t^
**Cognitive functions**			
Executive functions	14.38	2.76	0.28 **
Theory of mind	5.12	1.14	0.12
**Behavioral factors**			
Internalizing problems	48.39	10.87	0.16 ^t^
Externalizing problems	49.34	9.92	−0.03
**Familial factors**			
Parental stress	2.04	0.67	0.09
Quality of parent–child interactions	3.18	0.44	−0.11
Outcome			
Sociomoral reasoning	18.63	4.91	1

^t^*p* < 0.10 ** *p* < 0.01.

**Table 3:** Hierarchical regression analyses predicting sociomoral reasoning.

Predicting Factors	*R* ^2^	∆*R*^2^	*β*	*F*
SMR				
Step 1: Demographic characteristics	0.04	0.04		1.10
Sex			0.02	
Age at predictor assessment			0.01	
Parental education			0.17 ^t^	
Step 2: Cognitive functions	0.17	0.13 ***		3.67 **
Sex			−0.08	
Age at predictor assessment			−0.16	
Parental education			0.21 *	
Executive functions			0.38 ***	
Theory of mind			0.16 ^t^	
Step 3: Behavioral factors	0.21	0.04 ^t^		3.54 ***
Sex			−0.11	
Age at predictor assessment			−0.20 ^t^	
Parental education			0.17 ^t^	
Executive functions			0.37 ***	
Theory of mind			0.16 ^t^	
Internalizing problems			0.28 *	
Externalizing problems			−0.20 ^t^	
Step 4: Familial factors	0.21	0.01		2.86 **
Sex			−0.11	
Age at predictor assessment			−0.20 ^t^	
Parental education			0.16 ^t^	
Executive functions			0.38 ***	
Theory of mind			0.15	
Internalizing problems			0.27 *	
Externalizing problems			−0.22 ^t^	
Parental stress			0.07	
Quality of parent−child interactions			−0.01	

^t^*p* < 0.10 * *p* < 0.05 ** *p* < 0.01 *** *p* < 0.001.

## Data Availability

The data presented in this study are available on request from the corresponding author. The data are not publicly available due to ethical restrictions.
